# Selenoprotein P inhibits cell proliferation and ROX production in HCC cells

**DOI:** 10.1371/journal.pone.0236491

**Published:** 2020-07-31

**Authors:** Jianxin Wang, Pei Shen, Sha Liao, Lian Duan, Dandan Zhu, Jinling Chen, Liuting Chen, Xiaolei Sun, Yinong Duan

**Affiliations:** 1 Department of Laboratory Medicine, Affiliated Hospital of Nantong University, Nantong, Jiangsu, People’s Republic of China; 2 Department of Medical Informatics, School of Medicine, Nantong University, Nantong, Jiangsu, People’s Republic of China; 3 Department of Pathogen Biology, School of Medicine, Nantong University, Nantong, Jiangsu, People’s Republic of China; Hunter College, UNITED STATES

## Abstract

Selenoprotein P (SEPP1) is a kind of secretory glycoproteins with an antioxidant effect during the development of some diseases. In this study, we attempted to observe the expression of SEPP1 in livers from the patients with hepatocellular carcinoma (HCC) and explore its effect on HCC cells. All the tissues from patients with HCC were obtained from Affiliated Hospital of Nantong University. Western blot and immunohistochemical results showed that SEPP1 was reduced in HCC liver tissues. Its expression was negatively correlated with Ki67 expression in tissues. The expression of SEPP1 in normal liver cell line was significantly higher than those in the liver cancer cell lines. Serum starvation and release experiment demonstrated that SEPP1 expression was reduced and PCNA expression was increased, when the serum was re-added into cell culture system and the cells were on a proliferation state. After SEPP1 over-expression plasmid was transfected into HepG2 cells, cell proliferation of HepG2 cells and PCNA expression level were all inhibited by SEPP1. Results obtained via 8-isoprostane ELISA further indicated that inhibited ROS level was found in HepG2 cells transfected with SEPP1 over-expression plasmid. In addition, RT-qPCR results demonstrated that GPX1 expression levels increased in HepG2 cells transfected with SEPP1 over-expression plasmid. In conclusion, SEPP1 may inhibit the proliferation of HCC cells, accompanied by the reduction of ROS production and the increasing of GPX1 expression.

## Background

Hepatocellular carcinoma (HCC) is one of the most common cancers which could induce death worldwide. During the occurrence and development of HCC, multiple studies have demonstrated that oxidative stress plays an important role in promoting this progress [1, 2]. For example, hepatitis B virus (HBV) infection could induce the accumulation of mitochondrial reactive oxygen species (ROS), thereby inhibiting the expression of suppressor of cytokine signaling 3 (SOCS3) and activating the interleukin-6 (IL-6)/STAT3 pathway, ultimately leading to liver cancer [3]. Meanwhile, the developmental process of HCC is often accompanied by the continuous production of ROS, which can activate the NF-κB signaling pathway and promote the proliferation and migration of HCC cells [4].

Selenoprotein P (SEPP1) is a kind of secretory glycoproteins synthesized by the liver, and functions as the carrier of selenium and maintains the dynamic balance and distribution of selenium in the body [5]. Importantly, SEPP1 is also reported to have a strong antioxidant effect during the development of some diseases, including non-small cell lung cancer [6], inflammatory bowel disease [7] and so on. Xiao et al. have found that 4-ClBQ could induce oxidative stress in human skin keratinocytes HaCaT and overexpression of SEPP1 could suppress 4-ClBQ-induced oxidative stress and toxicity [8]. Both tumor necrosis factor-α (TNF-α) and H_2_O_2_ could inhibit SEPP1 expression in adipocyte 3T3-L1 cells and SEPP1 silencing could result in obviously oxidative stress and inflammation, accompanied by the increasing of inflammatory cytokines MCP-1 and IL-6 and the inhibition of adipocyte differentiation [9]. SEPP1 was also reported to be down-regulated in prostate cancer and result in the production of free radicals, thereby causing oxidative damage and promoting the development of prostate cancer [10].

Using the method of in situ hybridization, Li et al. reported that the expression of SEPP1 mRNA was significantly lower in HCC tissues than that in normal tissues [11]. Hence, they speculated that SEPP1 would participate in the occurrence and development of HCC. Importantly, TNF-α, which could induce oxidative stress in diverse cells, can suppress the promoter activity of SEPP1 in HepG2 cells [9, 12, 13]. In this study, we further observed the expression of SEPP1 in HCC patients and we also attempted to explore the mechanism by which SEPP1 could play the key role in inhibiting tumorigenesis of HCC.

## Materials and methods

### Patients

9 liver cancer specimens for western blot and 30 liver cancer specimens (including 11 cases of patients with poorly-differentiated tumors, 9 cases of patients with moderate-differentiated tumors, 10 cases of patients with well-differentiated tumors) for immunohistochemical experiment were all collected from inpatients with a clear pathological diagnosis in the Affiliated Hospital of Nantong University (March, 2016 to March, 2017). A total of 7 female cases and 23 male cases were included in this study. The collection of all the human tissues was approved by the Ethics Committee of Affiliated Hospital of Nantong University (Approval number: 2016030). The study didn’t involve the individual information of patients and the business interests during or after data collection. All data were fully anonymized before we accessed them. Briefly, the associated tissues were collected for the routine medical examination by pathologists after surgical removal by clinicians according to the clinical operation specification. Then we just obtained the residue tissues from the pathologists. Specimens for immunohistochemical experiment were paraffin-embedded, while specimens for western blot were directly preserved in -80°C.

### Cell lines and transfection

Human hepatoma cell line HepG2 (Catalog Number: SCSP-510) was purchased from Cell bank of Chinese Academy of Sciences (Shanghai, China). Human hepatic cell line (L02) and human hepatoma cell line (Huh7) were obtained from Jiangsu Province Key Laboratory for Inflammation and Molecular Drug Target, Nantong University [14]. All the cells were cultured in Dulbecco’s Modified Eagle’s Medium (DMEM, Gibco, USA) which was supplemented with 10% of fetal bovine serum (FBS). SEPP1 plasmid for overexpression of SEPP1 (SEPP1 BC015875.1) and control vector (EX-eGFP-M02) were all purchased from Genecopoeia (USA) and transfected into cells using Lipofectamine 2000 Transfection Reagent from Invitrogen (USA) according to its user manual. After 48 h cultured, cells were harvested for western blot, enzyme-linked immunosorbent assay (ELISA) for 8-isoprostane expression level detection and RT-qPCR.

### Western blot

Cells were transfected with or without plasmids and cultured in DMEM for 48 h. Proteins were collected from tissues or cells using RIPA Cell Lysis Buffer (Beyotime, China). Then 200 μg of protein was separated in SDS electrophoresis and transferred onto polyvinylidene fluoride (PVDF, Merck, Germany) membranes. After blocked with non-fat milk, the PVDF membranes were incubated overnight with primary antibodies (SEPP1, 1:100; PCNA, 1:100; Santa Cruz Biotechnology, USA) at 4°C, and subsequently with the secondary antibodies (Santa Cruz Biotechnology, USA) at room temperature for 2 h. Then the membranes were visualized using ECL reagents (Merck, Germany) under ChemiDoc XRS+ System (Bio-Rad, USA). Glyceraldehyde phosphate dehydrogenase (GAPDH, 1:1000, Goodhere, China) was used as the internal control.

### Flow cytometry

For cell cycle analysis, cells were serum-starved for 72 h in DMEM, and then the medium was replaced with DMEM containing 10% FBS. After cultured for indicated time, the cells were harvested and fixed with ethanol at 4°C overnight. Subsequently, the cells were incubated successively with RNase A (Sangon, China) at 37°C for 30 min and propidium iodide (PI, Biosharp, China) on ice for 30 min. Then cell cycle analysis was performed using a flow cytometer from BD Biosciences (USA).

### RT-qPCR

Cells were transfected with SEPP1 plasmid and cultured for 48 h. Total RNA was extracted from the associated cells using Trizol reagent (Invitrogen, USA) and 1 μg of RNA was used to reversal transcribe into cDNA using the kit from Thermo (USA). Then 100 ng of cDNA was used as the template for qPCR experiment using SYBR Premix Ex Taq Kit (Takara, Japan). The primers were synthesized by Sangon (Shanghai, China). All the experiments were repeated at least three times. The expression of the target gene was normalized to GAPDH and analyzed by the 2^-△△CT^ method.

### Immunohistochemistry

Paraffin-embedded sections were dewaxing by dimethylbenzene and treated by ethanol at various concentration gradient. Then the sections were heated in a pressure cooker in antigen retrieval solution. After treated with 3% H_2_O_2_, the sections were then blocked with donkey serum working solution. Then the sections were incubated with primary antibodies (SEPP1, 1:50; Ki67, 1:100; Santa Cruz Biotechnology, USA) at 4°C overnight and the associated secondary antibodies (Santa Cruz Biotechnology, USA) at 37°C for 1 h, followed by DAB color development. Then the sections were counterstained and dehydrated. After the sections were covered with cover slip, the images from the sections were acquired under Leica DM5000B microscope (Leica, Germany).

### ELISA for 8-isoprostane expression level detection

8-isoprostane competitive in vitro ELISA kit is purchased from Abcam (ab175819, USA). This kit is suit for the determination of 8-isoprostane (which was reported to be a best index of oxidative injury in a well-accepted oxidant stress rat model) expression in cells, serum and other biological samples [15, 16]. Cells were transfected with SEPP1 plasmid and cultured for 48 h. And the detection of 8-isoprostane expression in cells was performed according to the manual provided by Abcam (USA).

### MTT assay

For analysis of cell proliferation, cells transfected with various plasmids for indicated time points (0 h, 12 h, 24 h, 48 h) were harvested and treated with MTT (Sigma) for 4 h. Then DMSO was added and the absorbance was measured (570 nm) using the Bio-Tek ELISA reader (USA).

### Statistical analysis

All the experiments were repeated at least three independent times. The data were presented as the mean ± SEM and analyzed by the one-way ANOVA method or Independent Samples T-test by SPSS 17.0. P<0.05 represented that significant differences exist between the two compared groups.

## Results

### The expression of SEPP1 was low in HCC patients

**Fig 1:**
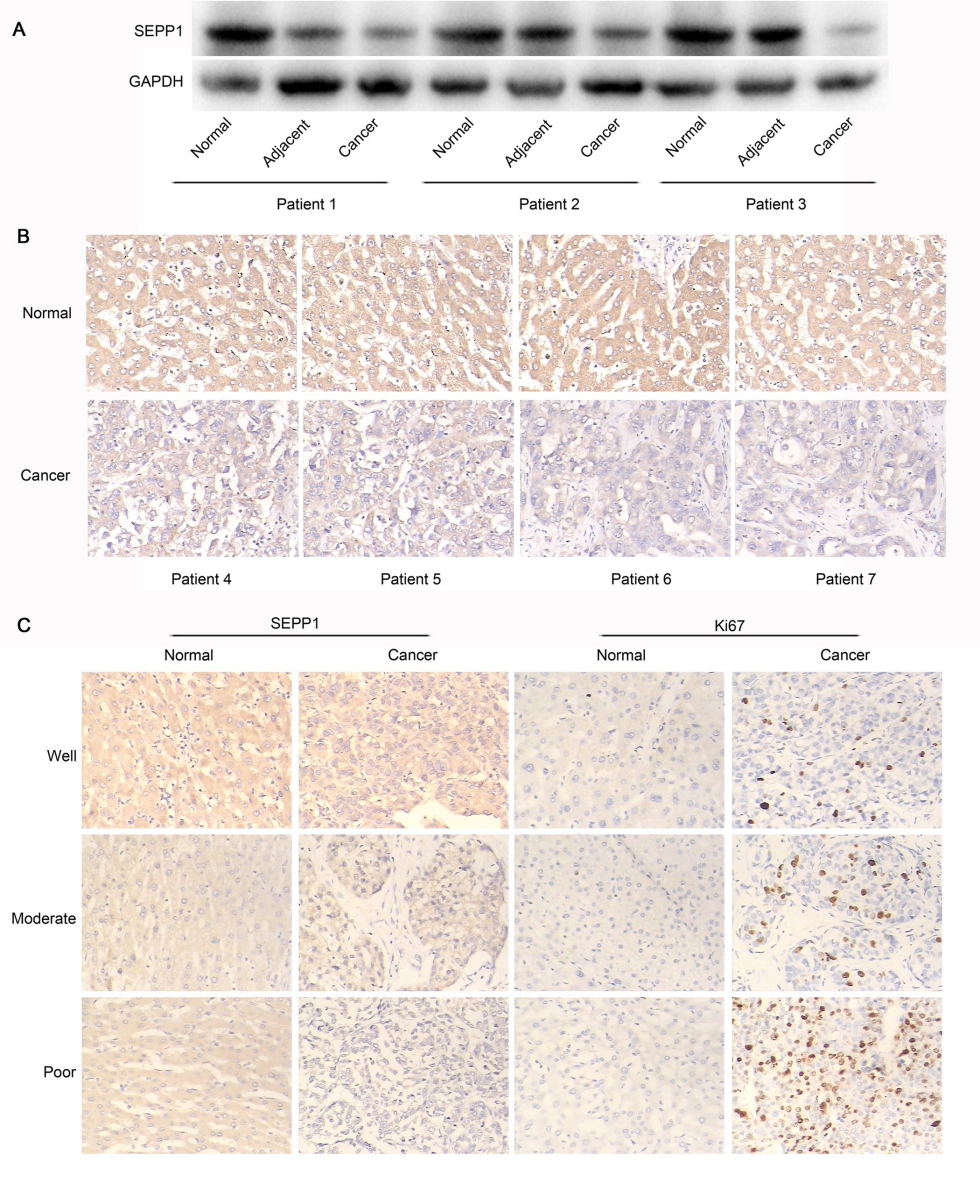
The expression of SEPP1 was low in HCC patients. A, The expression of SEPP1 in human tissues was detected by western blot. The expression of SEPP1 was low in cancer tissues. B, The expression of SEPP1 in human tissues was detected by immunohistochemistry. The expression of SEPP1 was low in cancer tissues. C, The expression levels of SEPP1 and Ki67 in human tissues with various degrees of differentiation were detected by immunohistochemistry. The poor degree of differentiation, the lower expression of SEPP1 and the higher expression of Ki67.

Previously, Li et al. demonstrated that the expression of SEPP1 mRNA was significantly lower in HCC tissues than that in normal tissues by the method of in situ hybridization [11]. In this present study, we found that the expression of SEPP1 protein was also down-regulated in HCC tissues, compared to that in the adjacent tissues and in the normal tissues (Fig 1A). Similarly, the results obtained from immunohistochemical method were also indicated that SEPP1 protein showed low expression in HCC tissues, while SEPP1 protein showed high expression in normal tissues (Fig 1B). Hence, both the results obtained from Li et al. and us were all demonstrated that the expression of SEPP1 was low in HCC patients. Which was more importantly, the immunohistochemical results (Fig 1C) also showed that a negative correlation existed between SEPP1 and Ki67 expression. Since Ki67 was reported to be a traditional proliferation and differentiation index in numerous tumor [17], we demonstrated that the poor degree of differentiation, the lower expression of SEPP1. In addition, data (HTSeq-FRKM) from 374 of liver hepatocellular carcinoma (LIHC) cases and 50 of normal human were also obtained and analyzed by using the Cancer Genome Atlas Program (TCGA, https://www.cancer.gov/about-nci/organization/ccg/research/structural-genomics/tcga) and R3.6.1. The results also showed that SEPP1 expression in LIHC cases was lower than that from normal human group (LogFC = -0.9006, P = 1.647*10^−14^, S1 Fig).

### The expression of SEPP1 was low in proliferating HepG2 cells

**Fig 2:**
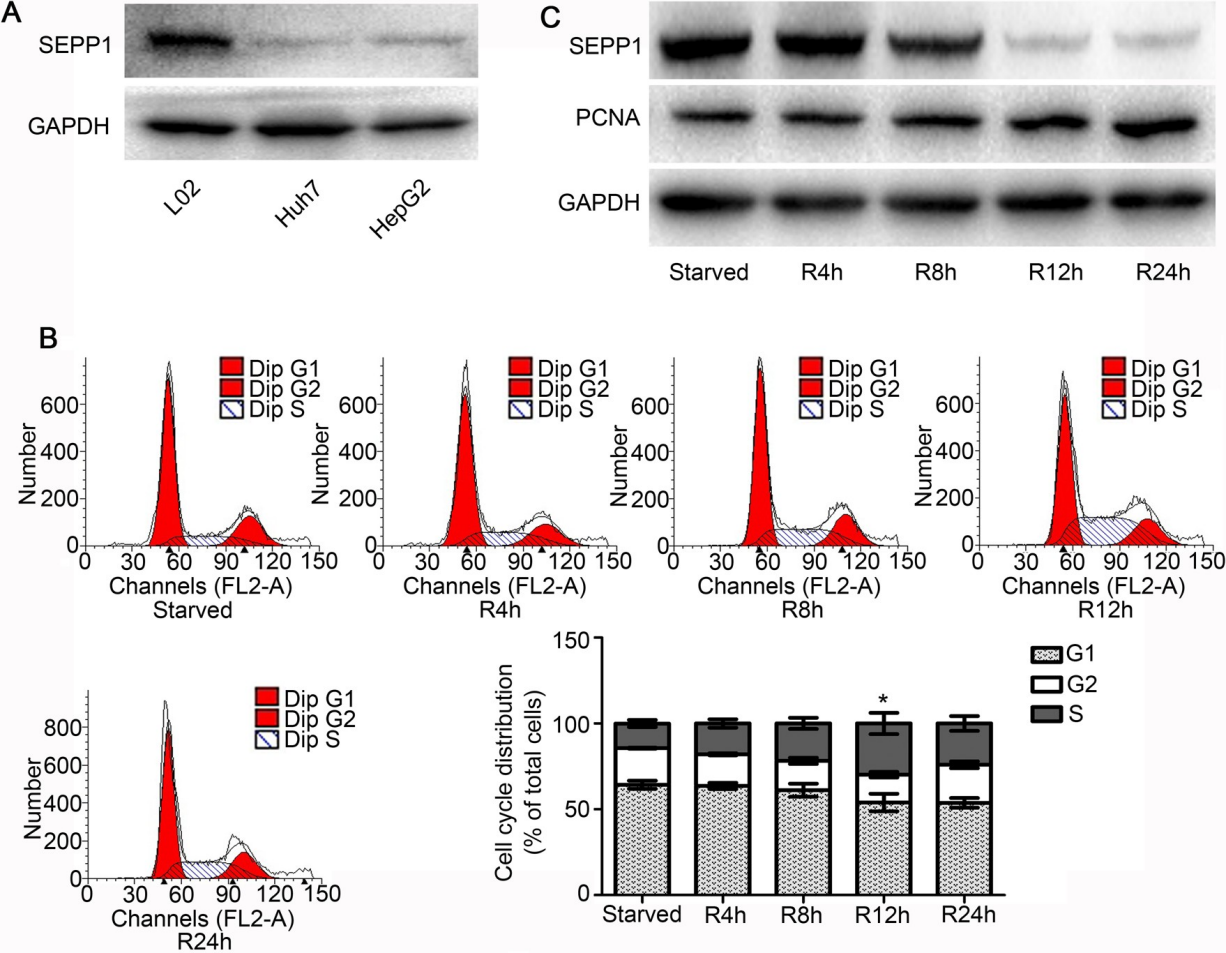
The expression of SEPP1 was low in proliferating HepG2 cells. A, The expression levels of SEPP1 in L02, Huh7 and HepG2 were detected by western blot. The expression of SEPP1 was lower in HepG2 cells and Huh7 cells than that in L02 cells. B, Cell cycles of HepG2 under serum-starved condition or refeeding with serum for various hours were analyzed by flow cytometry. HepG2 cells were in the proliferation state when the cells were re-added with FBS. *P<0.05, compared to serum-starved group. C, The expression levels of SEPP1 and PCNA in HepG2 under serum-starved condition or refeeding with serum for various hours were detected by western blot. The expression of SEPP1 was low in proliferating HepG2 cells.

In order to observe the expression of SEPP1 in proliferating HCC cells, we firstly observed SEPP1 expression in L02, Huh7 and HepG2 cells. The results showed that SEPP1 expression was low in HCC cell lines, while it was up-regulated in human hepatic cell line L02 cells (Fig 2A). Then we used HepG2 as the objective of the next study. As the results shown in Fig 2B, cell cycle of HepG2 cells was arresting at G1 phase when the cells were under serum starvation condition for 72 h. After cell cycle synchronized successfully by serum starvation, 10% FBS were then re-added into HepG2 cells and the cells were gradually transformed from G1 phase to S phase, at which time HepG2 cells were in the proliferation state (Fig 2B). Moreover, we further confirmed that the expression of SEPP1 was at a high level in HepG2 cells synchronized by serum starvation for 72 h (Fig 2C). However, the expression of SEPP1 began to decrease gradually as serum release time goes on. On the contrary, the expression of PCNA, a proliferation indicator, gradually increased after 4 h of serum release (Fig 2C), indicating that the cells were in a state of proliferation. Hence, the above phenomenon suggested that the expression of SEPP1 might be closely related to the proliferation of HCC cells.

### Overexpression of SEPP1 in HepG2 cells could inhibit the proliferation of HepG2 cells

**Fig 3:**
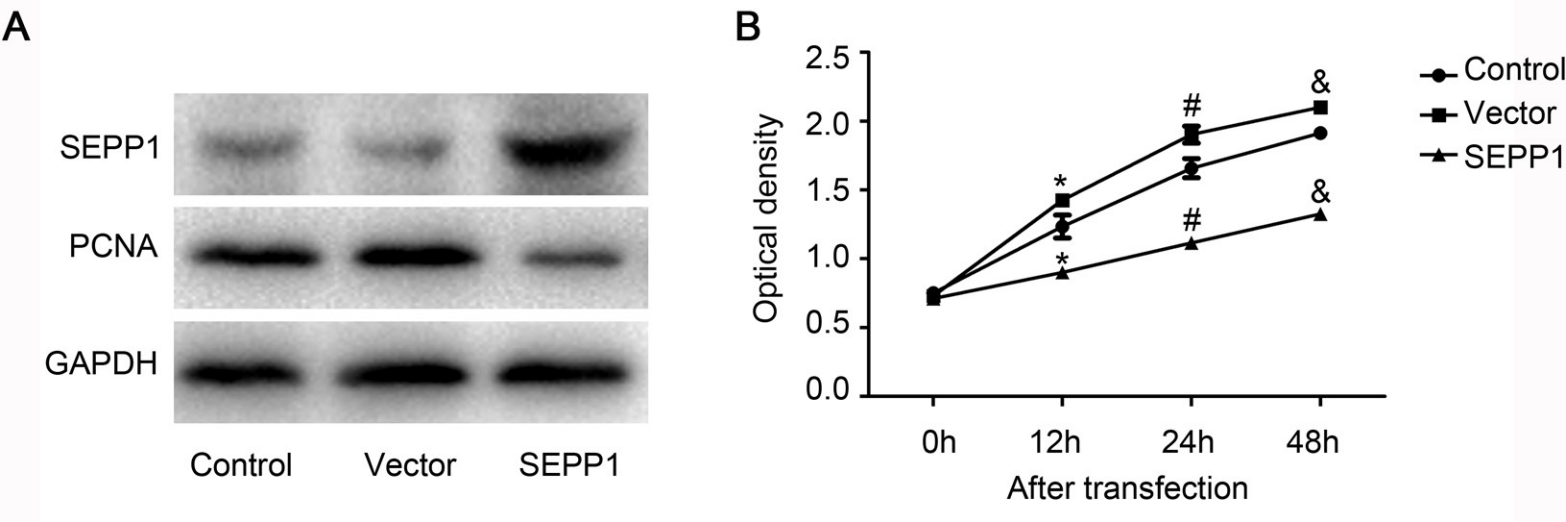
SEPP1 in HepG2 cells could inhibit the proliferation of HepG2 cells. A, The expression levels of SEPP1 and PCNA in HepG2 cells transfected with various plasmids for 48 h were detected by western blot. Over-expression of SEPP1 could inhibit PCNA expression in HepG2 cells. B, Cell proliferation of HepG2 cells transfected with various plasmids for indicated times (0 h, 12 h, 24 h, 48 h) was analyzed by MTT assay. Over-expression of SEPP1 could suppress proliferation of HepG2 cells. *, #, &P<0.05, compared to each control group (transfected with PBS).

To observe the effect of SEPP1 on the proliferation of HepG2 cells, SEPP1 plasmid for overexpression was then transfected into HepG2 cells. The data in Fig 3A confirmed that SEPP1 expression was up-regulated in HepG2 cells transfected with SEPP1 plasmid for 48 h. The results of Fig 3A also demonstrated that overexpression of SEPP1 could induce the reduction of PCNA expression. In addition, results obtained by MTT method also proved that compared to each control groups, the proliferation of HepG2 cells was significantly inhibited in the cells transfected with SEPP1 plasmid (Fig 3B, P<0.05). Hence, all the above results suggested that SEPP1 may inhibit the growth of HCC by inhibiting the proliferation of HCC cells.

### Overexpression of SEPP1 in HepG2 cells could inhibit the production of ROS and induce the expression of GPX1 in HepG2 cells

**Fig 4:**
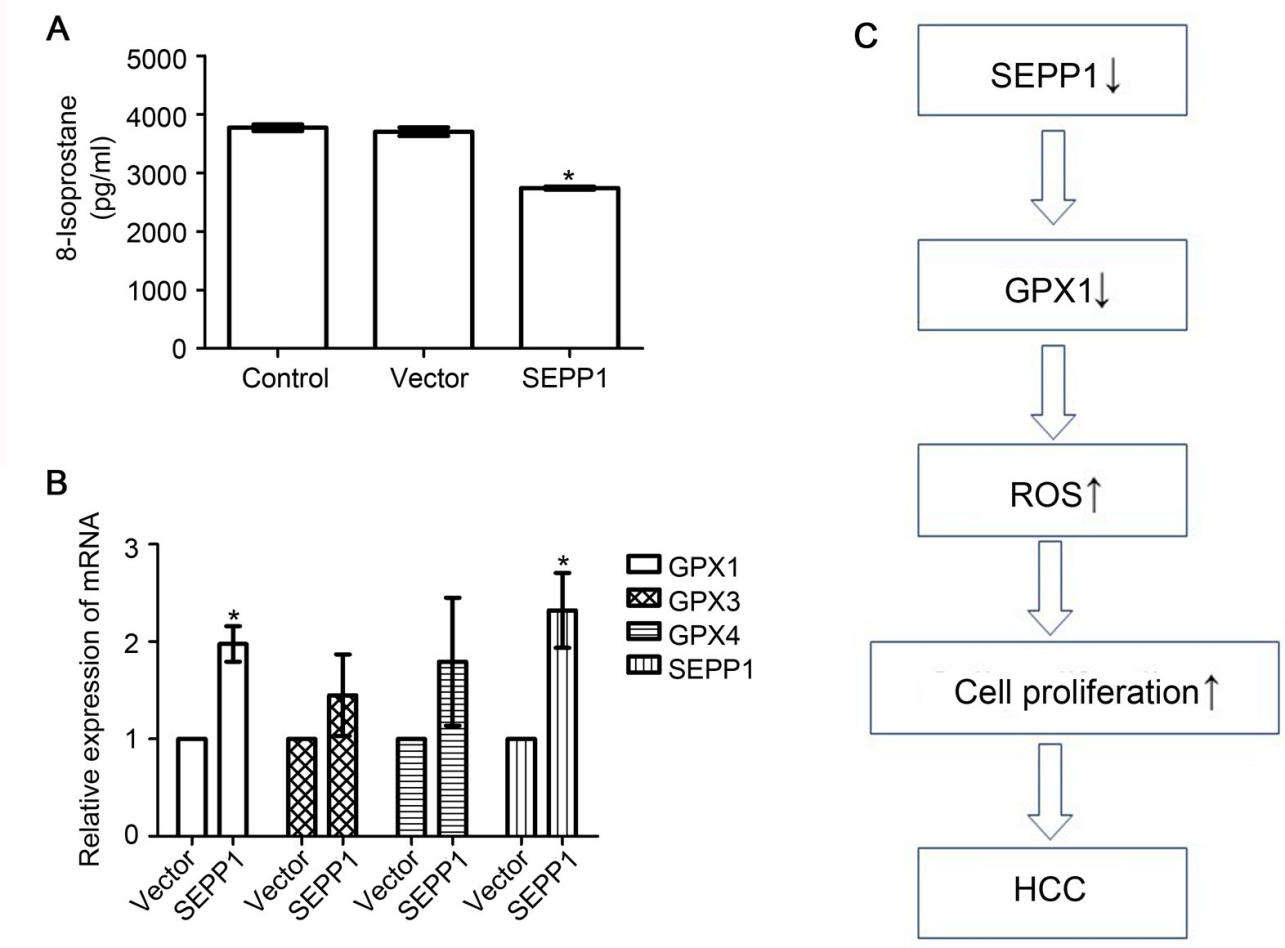
SEPP1 could inhibit ROS production and induce GPX1 expression in HepG2 cells. A, The expression level of 8-isoprostane in HepG2 cells transfected with various plasmids for 48 h were detected by ELISA. Over-expression of SEPP1 could inhibit ROS production in HepG2 cells. *P<0.05, compared to control group (transfected with PBS). B, Expression of GPXs in HepG2 cells transfected with various plasmids was for 48 h analyzed by RT-qPCR. Over-expression of SEPP1 could up-regulate GPX1 expression in HepG2 cells. *P<0.05, compared to control group (transfected with vector). C, Model of the potential relationship among SEPP1, ROS and GPX1 in HCC cells.

Since ROS was reported to promote the proliferation of HCC cells [4], we then attempted to observe the effect of SEPP1 on ROS production in HepG2 cells. We used ELISA method to detect the expression level of 8-isoprostane (an indicator of ROS) in the cells [15]. Results in Fig 4A showed that the content of 8-isoprostane in SEPP1 overexpression group was significantly lower than those both in negative control group and empty vector group (P<0.05). SEPP1 can effectively restrain the generation of ROS in HCC cell lines HepG2. Glutathione peroxidases (GPXs), which belong to selenocysteine-containing redox enzymes, are the important antioxidant enzymes for decreasing ROS-induced damage in human body [18]. Hence, we observed that if SEPP1 could also affect the GPXs content in HepG2 cell line in subsequent studies. Our results showed that overexpression of SEPP1 could effectively up-regulate the mRNA expression level of GPX1, but not GPX3 and GPX4 in HepG2 cells (Fig 4B, P<0.05). Hence, all the above results indicated that SEPP1 may inhibit the growth of HCC by regulating the ROS level in HepG2 cell line (Fig 4C).

## Discussion

SEPP1 was the main supplier of selenium in human body and it was reported to regulate cell growth of tumor cells via modulating cell proliferation, apoptosis, differentiation, and so on. Previous study of Meyer et al. demonstrated that both the serum concentrations of SEPP1 and selenium in patients with renal cancer were lower than those in normal human [19]. The expression of SEPP1 was negatively correlated to the mortality of renal cancer [19]. Calvo et al. found that expression levels of SEPP1 were all down-regulated in human prostate carcinomas and in prostate cancer cell lines compared with that in normal prostate [20]. In addition, SEPP1 expression was significantly reduced in colon cancers, while its expression was abundant in normal colon mucosa [21]. In our present study, we also found that the expression of SEPP1 protein was down-regulated in HCC tissues, while SEPP1 protein showed high expression in normal tissues. This result was consistent with that obtained from TCGA database (S1 Fig). Meanwhile, the expression levels of SEPP1 in human HCC cell lines (HepG2 cells and Huh7 cells) were all lower than that in human hepatocyte cell line (L02 cells). Our results were also similar with those reported by Li et al., who confirmed that the expression of SEPP1 mRNA was significantly lower in HCC tissues and in liver cirrhosis tissues [11]. Which was more importantly, we also confirmed that the expression of SEPP1 in HCC was negatively correlated with the expression of Ki67, which represented proliferation of tumor cells and tumor metastasis in HCC. In addition, experiments in vitro further proved that SEPP1 expression was negatively correlated to PCNA expression. Over-expression of SEPP1 could inhibit PCNA expression in HepG2 cells, as well as cell viability of HepG2 cells. Hence, all the results above showed that SEPP1 may be involved in regulating cell proliferation of HCC cells.

More and more studies were focused on the antioxidant capacity of SEPP1 in occurrences and development of various diseases. For example, Traulsen et al. have demonstrated that SEPP1 could inhibit CuCl_2_-induced low-density lipoproteins (LDL) oxidation [22]. Eckers et al. also found that SEPP1 could reduce radiation-induced accumulation of ROS in NHFs cells and protect NHFs from radiation-induced normal cell injury [23]. In addition, loss of SEPP1 could increase ROS production and DNA damage in epithelial cells via WNT signaling [7]. Since oxidative stress also promotes the development of HCC [1, 2], we wonder if SEPP1 could exhibit its antioxidant capacity in HCC. As expected, we found that over-expression of SEPP1 could obviously suppress the ROS production in HepG2 cells. The results reported by Yi et al. also proved that human hepatitis B virus X protein (HBx) could induce lipid peroxidation and block SEPP1 expression in HepG2 cells [24]. In addition, TNF-α could also induce oxidative stress and inhibit the promoter activity of SEPP1 in HepG2 cells simultaneously [12, 13, 24].

GPXs were the important antioxidant enzymes for decreasing ROS-induced damage in human body [18]. It was established that GPXs play essential roles in the development of various tumors. For example, GPX3 could suppress the development of colitis-associated carcinoma via inhibiting ROS production [25]. In human HCC cell lines, selenium could promote GPX4 expression and then inhibit lipid peroxides and the expression of IL-8 and vascular endothelial growth factor (VEGF) [26]. Importantly, Meplan et al. mentioned that SEPP1 and GPXs could modulate the risk of breast cancer [27]. They demonstrated that GPX1 had the ability to respond to ROS to alleviate the tumor progression of breast cancer and supplemental of Se could maintain the activity of GPX [27]. Zhou et al. also confirmed that estrogen could up-regulate both hepatic GPX1 and SEPP1 expression in OVX rats [28]. SEPP1 could also increase the enzymatic activities of GPX and protect the endothelial cell line Ea.hy926 cells from oxidative damage [29]. In our present study, we observed the expression of GPX1, GPX3 and GPX4 in HepG2 cells transfected with SEPP1 and the results demonstrated that over-expression of SEPP1 in HepG2 cells could induce GPX1 expression in HCC cell lines.

## Conclusion

In conclusion, the expression of SEPP1 is low in liver cancer tissue and it is negatively correlated with Ki67. SEPP1 may inhibit proliferation of HCC cells by reducing the production of ROS and promoting the production of GPX1 in HCC cells (Fig 4C).

## Supporting information

S1 Fig The expression of SEPP1 in HCC (data from TCGA). There are 374 patients with LIHC in tumor group and 50 normal human in normal group. The data and figure were obtained and analyzed by using the Cancer Genome Atlas Program (TCGA) and R3.6.1.
(TIFF)

S1 Raw images (ZIP)

## Abbreviations

GPXs: Glutathione peroxidases
HBV: hepatitis B virus
HCC: hepatocellular carcinoma
LDL: low-density lipoproteins
NSCLC: non-small cell lung cancer
ROS: reactive oxygen species
SEPP1: Selenoprotein P
SOCS3: suppressor of cytokine signaling 3
TNF-α: tumor necrosis factor-α
VEGF: vascular endothelial growth factor
