# Supplementary figures and images for: Selenoprotein P inhibits cell proliferation and ROX production in HCC cells
Source: PLoS One. 2020 Jul 31;15(7):e0236491. doi: 10.1371/journal.pone.0236491 (PMC7394388; doi:10.1371/journal.pone.0236491)

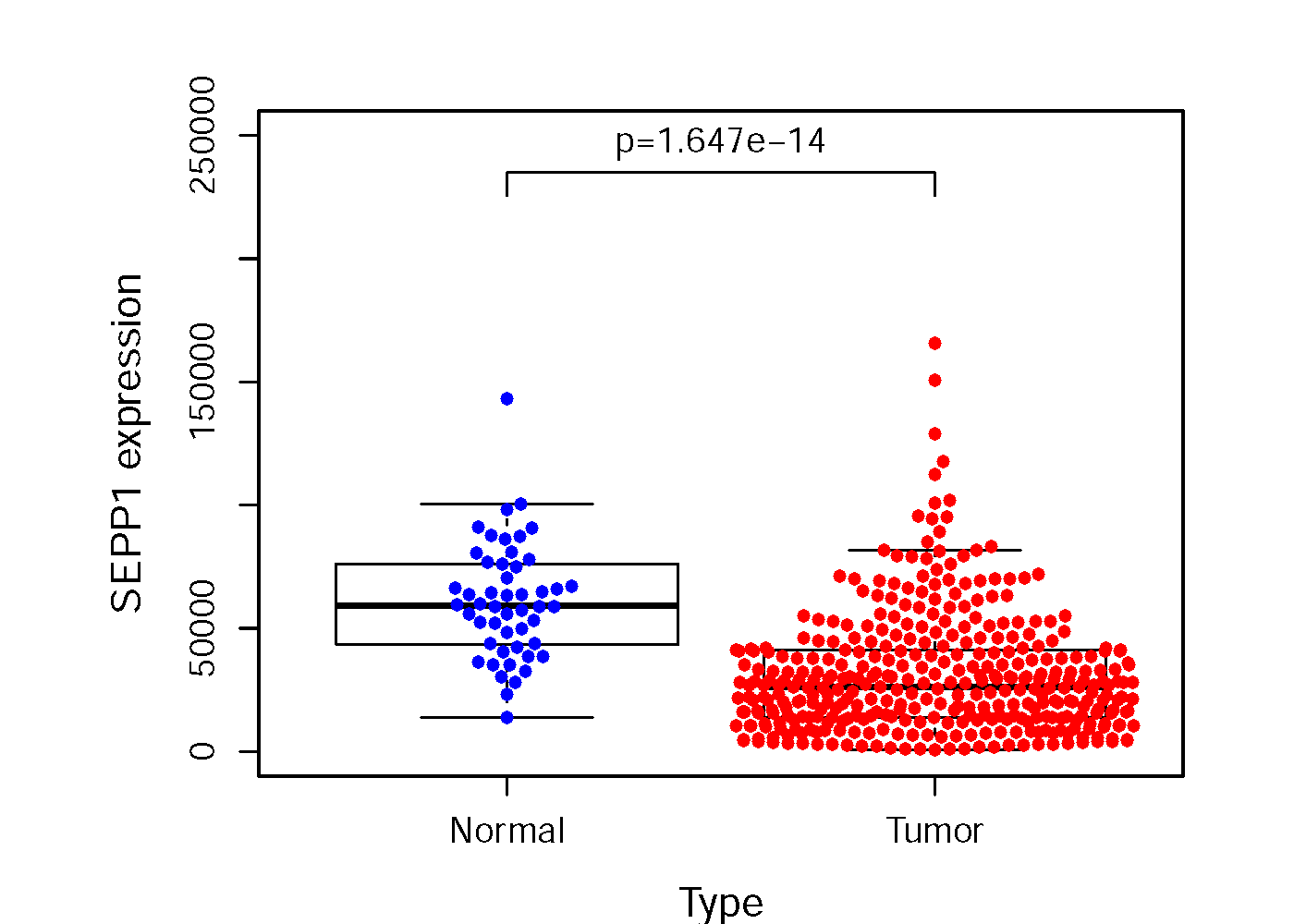

Supplement: S1 Fig — There are 374 patients with LIHC in tumor group and 50 normal human in normal group. The data and figure were obtained and analyzed by using the Cancer Genome Atlas Program (TCGA) and R3.6.1. (TIFF) [file pone.0236491.s001.tiff]
